# Evaluating the sensitivity and specificity of a severe sepsis tool utilized at a community hospital in Miami, FL

**DOI:** 10.1186/cc14019

**Published:** 2014-12-03

**Authors:** J Hirigoyen

**Affiliations:** 1 4 Tower Medical-Surgical Unit, Baptist Hospital of Miami, Miami, FL, USA

## Introduction

Since the initial development of the Surviving Sepsis Campaign guidelines outlining the management of severe sepsis, there has been an absolute discount on the management of septic patients in medical surgical units. In efforts to improve severe sepsis, a community hospital in Miami adopted a severe sepsis screening tool (SSST) to rapidly identify severe septic patients in medical surgical units. A pilot study was conducted to evaluate the sensitivity and specificity of the SSST.

## Methods

**Figure 1:**
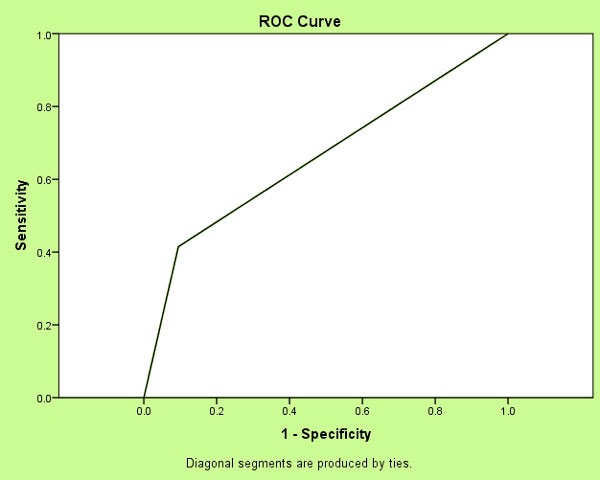
**ROC curve**.

A descriptive retrospective study. There were two phases. Phase 1 evaluated the percentage of patients with sepsis criteria utilizing the SSST. Patients admitted to 4 Tower during 2013 presenting with a diagnosis of sepsis syndrome and admitted to 4 Tower presenting without sepsis syndrome were reviewed. Phase 2 evaluated the sensitivity and specificity of SSST from August 2013 to January 2014. Total number of patients admitted to 4 Tower: of those patients, total number with discharge diagnosis of sepsis, total number who screened positive >1 time during hospital stay, and total number who screened negative during hospital stay; there were five missing cases. The receiver operating curve (Figure 1) and the respective area under the curve were calculated. Utilizing a 2 × 2 design, the sensitivity and specificity of the tool was calculated.

## Results

Phase 1: a total of 220 patients records were reviewed, a frequency distribution was utilized (Table 1), demonstrating that the SSST identified those patients with sepsis criteria 76 % (*n *= 167) of the time. Phase 2: a total of 1,555 patients were included during phase 2. A 2 × 2 design (Table 2) was utilized: 78 patients were identified as true positive and 1,233 patients were identified as true negative. The study yielded a sensitivity of 41.49% and a specificity of 90.53%. The positive predictive value of the tool was estimated at 37.68%, negative predictive value was estimated at 91.81% and disease prevalence was 12.13%. Area under the receiver operating curve (Table 3) was 0.66.

**Table 1:** Frequency distribution.

Statistics	Diagnosis on admission	Sepsis tool identifies sepsis		
Valid	220	220		
Missing	0	0		

**Frequency table**	**Frequency**	**Percent**	**Valid percentage**	**Cumulative percentage**

Diagnosis on admission				
Valid	220	100.0	100.0	100.0
Sepsis tool identifies sepsis				
Not valid	53	24.1	24.1	24.1
Valid	167	75.9	75.9	100.0
Total	220	100.0	100.0	

**Table 2:** The 2 × 2 design.

	Sepsis present	Sepsis absent
Positive	78 true positive	129 false positive
Negative	110 false negative	1,233 true negative

**Table 3:** Area under the curve.

Test result variable(s):screen
Area
0.660

## Conclusion

A two-phase retrospective chart review study demonstrated that the SSST utilized at a community hospital in Miami had a sensitivity value of 41.49% and a specificity value of 90.53% when evaluating medical surgical patients. These results indicate the tool is accurate in detecting patients that are not septic; however, it is not reliable in identifying patients who are truly septic. Further studies need to be conducted to validate the sensitivity and specificity of the SSST; changes will be recommended in an effort to improve sensitivity.

